# A comparison of rural–urban differences in out-of-pocket expenses among older Mexicans with diabetes

**DOI:** 10.3389/fpubh.2022.1025159

**Published:** 2022-10-21

**Authors:** Alfonso Rojas Alvarez, Christian E. Vazquez, Mariana Lopez-Ortega, Jacqueline L. Angel

**Affiliations:** ^1^Lyndon B. Johnson School of Public Affairs, University of Texas at Austin, Austin, TX, United States; ^2^School of Social Work, University of Texas at Arlington, Arlington, TX, United States; ^3^Instituto Nacional de Geriatría, Mexico City, Mexico

**Keywords:** diabetes, out of pocket cost, public health, chronic diabetes, rural urban difference

## Abstract

**Objective:**

To compare total out-of-pocket expenses for physician visits and medications among older adults living with diabetes in Mexico from urban, semi-urban, and rural areas.

**Methods:**

The sample included 2,398 Mexicans aged 65 years and older with self-reported diabetes from the 2018 Mexican Health and Aging Study. Out-of-pocket expenses for physician visits and medications were regressed on locality, controlling for several factors.

**Results:**

The profile of those with higher out-of-pocket medication expenditures included rural localities, higher education, unmarried, depressive symptoms, participation in Seguro Popular, and lacking insurance. In the multivariate analysis, rural older adults with diabetes paid a higher amount in medication expenditures compared with other localities.

**Conclusion:**

Differences in locality are closely tied to the effective implementation of Seguro Popular. Although this program has improved access to care, participants have higher out-of-pocket expenditures for medications than those on employer-based plans across all localities. Among all groups, the uninsured bare the highest burden of expenditures, highlighting a continued need to address health inequities for the most underserved populations.

## Introduction

In 2016, Mexico declared the increase in diabetes a public health emergency (1). Currently, the disease affects roughly 14% of the population and is becoming the nation's leading cause of death and disability. Even during the COVID-19 pandemic, diabetes was the third cause of death, the leading cause of death for adults aged between 45 and 64 years, and the second leading cause of death for adults aged 65 years and older (2). According to a recent 12-year study (3), death rates were approximately four times as high among people with diabetes than among those without diabetes. Diabetes care has been identified as both costly and intricate, for families and policymakers alike (4). It has been reported that households with at least one household member with chronic disease face health expenditures that are 25–34% higher than households without such individuals (5, 6). Comparing the economic impact of diabetes-related healthcare costs in 2009 vs. 2011, researchers found a 33% increase in financial requirements for both insurers and consumers (total costs of over $775 million) 0.6. In addition to direct costs such as medications, consultations, and hospitalizations, there are also indirect costs due to early deaths and disability (7). Given the nation's aging population, estimates indicate the prevalence of diabetes will grow in the coming decades, reaching 18% and 22% of the total population by 2030 and 2050, respectively (8).

Evidence suggests that urban and rural dwellers will not bear the burden of increases in diabetes evenly, as healthcare access and expenditures vary sharply between these two groups in Mexico (9). Over the last 30 years, the rates of healthcare coverage increased from 30 to nearly 50% in rural areas of Mexico, decreasing the gap in the rate of healthcare utilization between individuals in urban (12.1%) and rural (9.9%) areas (10). Though, a systematic review found that people living in rural areas continue to face barriers to care such as lack of resources with which to afford treatment (including medication), low literacy, and low continuity among healthcare professionals in rural areas. Other studies suggest that people living in rural areas tend to have poorer healthcare access and poor quality of care and that not having insurance coverage was associated with a lower likelihood of both overnight visits in the hospital and visiting a physician (11). Compared with urban households, rural households are also at elevated risk of catastrophic health expenditures from medication expenses (12).

Previous studies have explored the differentiated costs of healthcare between rural and urban locations in different countries, such as China and Mexico (13, 14). In the latter, due to barriers such as lack of access to care, lower medical appointments and hospitalization rates, as well as lower health insurance rates than in urban, and even semi-urban areas, rural dwellers may face higher out-of-pocket healthcare expenditures than the general population (11, 15). Managing diabetes care in rural areas is particularly difficult because of overall health and socioeconomic differences among older adults. Mexican rural dwellers are more likely to be older, belong to indigenous populations, and be migrants than the general population, factors that exacerbate inequalities in access to healthcare and expenditures (10). Mexico has a large informal economy, especially in rural areas, leading to an increased risk of lack of insurance coverage (16). Given that previous studies that have examined the relationship between out-of-pocket expenses among individuals with diabetes from rural, semi-urban, and urban areas in Mexico are more than a decade old, there is a clear need for studies that update this information.

The literature offers insights into other factors, in addition to locality, that may impact out-of-pocket healthcare expenditures for people living with diabetes. For example, older age is linked to a higher prevalence of diabetes and higher diabetes-related healthcare expenses (17, 18). Evidence suggests the gender gap in aging and rural areas generates systemic inequities, perpetuating worse outcomes for women, in the treatment of diabetes (19, 20). Diabetes reduces labor market participation, which decreases access to health insurance, increasing medical expenses (21). A report by the Mexican Ministry of Health found that people with lower education levels are at higher risk of diabetes and that the effects of education are greater for women (22). While educational attainment increased steadily throughout the twentieth century in Mexico, educational opportunities and quality of education in rural areas have consistently lagged urban areas (23).

Certain health risk factors are also associated with increased or decreased expenditures among people with diabetes. High rates of comorbidity of depression and diabetes have been reported, potentially leading to more hospital visits or need of more medications. Similarly, smokers tend to have higher rates of diabetes, which can also lead to more hospital visits and other comorbidities (24). Adherence to and use of medications due to poor health is also associated with higher medical expenses. For example, a systematic review examining the cost of medication adherence and persistence found that medication non-adherence increased healthcare costs for individuals; while it lowered medication costs, other costs more than offset such savings (25).

Perhaps, the greatest factor impacting personal healthcare expenditures is insurance coverage, including type and lack of coverage (26). A qualitative study in four rural communities in the state of Puebla, Mexico, found severe limitations in access to public services that lead to individuals seeking private care where expenses were almost exclusively out of pocket (27). Other studies support this, suggesting that in Mexico, being older and living in rural areas is associated with not having health insurance (16).

The structure of the healthcare system in Mexico shapes out-of-pocket expenses. People working in the formal labor market are insured under mandatory social security insurance through either the Mexican Institute of Social Security (IMSS) for private sector employees or the Institute of Social Security of State Workers (ISSSTE) for federal- and state–local-level public servants. Some publicly owned companies such as Mexico's state oil company, Pemex, and the Armed Forces have their own sub-system that provides healthcare coverage and social security benefits to members and employees. The government introduced Seguro Popular de Salud (People's Health Insurance, commonly called Seguro Popular) in 2003 to address out-of-pocket expenditures which had been very high (28, 29). Under Seguro Popular, healthcare services for the uninsured are provided by the Ministry of Health through the Social Protection in Health System (30). Substantial portions of the Mexican population aged 50 years and older, 49% of men and 45% of women, lacked health insurance in 2001; these numbers had shrunk to 17% and 14%, respectively, by 2012 (31). Seguro Popular met its goal of increasing coverage for the uninsured, those in the informal labor market, and the self-employed (29). Of the total rural population aged 50 years and older that were uninsured in 2001, almost 50% had insurance by 2012 (31). This gain was relatively lower in urban areas (20%). Disparities in access to care and cost continued based on insurance coverage and services available even after the program's introduction (10).

Seguro Popular was replaced in 2020 by the Health Institute for wellbeing (INSABI) as part of an effort to provide universal public and free at the point of service healthcare; however, the current study draws on data gathered before the transition, which remain relevant because the factors that affected disparities persist. This study provides context as researchers continue to monitor the impact of the transition, especially as the new program shares several features with the old one.

The objective of this article was to understand locality (rural, semi-urban, and urban) differences in out-of-pocket health expenses for older adults with diabetes. We build on previous research to estimate the associations of the known underlying factors on medical visit and prescription drug out-of-pocket expenditures by focusing on differences for older adults in rural, semi-urban, and urban areas (16, 31). This study contributes to the literature by utilizing a unique set of demographic, health risk, and health insurance coverage variables to account for the larger context in which older adults experience healthcare. We hypothesize that these expenditures are likely to be higher in rural areas than in semi-urban and urban areas based on the literature which suggests older adults from rural areas tend to experience worse overall health outcomes and lower health insurance which would provide treatment options with less individual expenditures.

## Data and methods

The study employs the fifth wave of the Mexican Health and Aging Study (MHAS, 2018; *N* = 17,114) (32), a nationally representative household survey of Mexican adults aged 50 years and older. The analytic sample began with 4,062 individuals who reported a diabetes diagnosis and were above 65 years of age, and then, 2,368 respondents had complete information on the study variables. A sensitivity analysis was conducted to assess differences from the starting analytic sample (4,062) and the complete cases (2,368). In the complete case sample, the main differences were that the rural population had a higher percentage of women and completed less years of formal schooling compared with the urban and semi-urban samples. Given the minor differences in the samples, the final sample included only those 2,368 individuals without missing data for all variables used in the models.

## Measures

### Dependent variables

We use two key variables to measure out-of-pocket expenses: *medication costs* and *medical visit costs*. All expenditures are measured in Mexican pesos. For *medication costs*, the corresponding question in the survey asked, “*Think about the last year, in a typical month how much was paid for the medications you take?*”, while for *medical visit costs*, the question was related to the last year and asked “*Including all visits, how much did you pay for these services?—Medical visits*.” These costs are self-reported in MHAS in pesos and include all expenditures, whether they are used to cover diabetes treatment or not. Both variables are transformed to log to account for non-linearities in the distribution of the responses, which can be illustrated from the high standard deviation in Table 1. Given that the dependent variables are presented in log form to account for non-linearities in expenditures, to interpret the regression coefficients, the reported value will be exponentiated, and the resulting number will represent the percent change in either medication expenses or medical visit expenses, per one unit variation in each measurement.

**Table 1:** Summary Demographics (% or mean/SD), adults aged 65 Years or older who have been diagnosed with diabetes (*n* = 2,368), 2018 Mexican health and aging study).

	**Rural**	**Semi-urban**	**Urban**
Proportion	13.2%	24.3%	62.5%
Age	63.48/8.26	64.95/9.15	65.69/9.23
Female respondents	65%	62%	59%
Married	60%	48%	49%
Employed	32.5%	32.5%	35%
Educational attainment (in years)	5.60/3.75	6.66/3.87	7.88/4.25
Depression	38%	41%	35.6%
Smoker	28.3%	34.1%	42.5%
Diabetes medication	91.9%	91%	91.8%
Insulin	23%	27%	29%
Social security institution	46%	64%	82%
No insurance	3.8%	3%	3.3%
Seguro Popular	52.7%	38%	15%
No medical visits	13.8%	14.4%	15.1%
Medical visit costs (in pesos)	1,402/7,100	1,047/4,944	1,151/6,202
Medication costs (in pesos)	839/3,594	846/5,968	751/4149
N	311	577	1,480

*SD* standard deviation.

### Independent variables

Individual determinants of out-of-pocket expenses were expressed in several categories. First, locality was classified as three categories: urban (population > 100,000), semi-urban (between 25,000 and 100,000), and rural (< 25,000). These groupings were based on categorizations employed by previous studies and are useful given the structural differences that exist in semi-urban localities compared with both urban and rural groups in terms of availability of services, travel distances, and the economic activities predominant in these areas (33).

A second group of independent variables represented *demographic characteristics*: (1) *age* of respondent in years; (2) *female respondents*, coded 1 if the respondent identified as female and 0 for male; (3) *married*, coded 1 if the respondent identified as married and 0 otherwise (single, divorced, separated from a civil union, widowed from a civil union); (4) *employed*, coded 1 if the respondent indicated they are currently working and 0 if they report being retired, looking for work or not working; and (5) *educational attainment*, reported as number of years of school completed by the respondent.

Third, *health* variables consisted of: (1) *depression symptoms*, coded 1 if the respondent reported having felt depressed and 0 if not; (2) *smoking*, coded 1 if the respondent reported smoking and 0 if not; (3) *diabetes medication*, coded 1 if the respondent reported taking oral medication for diabetes and 0 if not; and (4) *insulin*, coded 1 if the respondent reported taking insulin and 0 if not.

Fourth, *healthcare system* variables included: (1) *social security institution*, coded 1 if the respondent reported being affiliated with an employment-based institution, such as IMSS, ISSSTE, Pemex, the military, or any others and 0 otherwise; (2) *no insurance*, coded 1 if the respondent reported not having any sort of insurance and 0 otherwise; (3) *Seguro Popular*, coded 1 if the respondent reported being affiliated with Seguro Popular and 0 otherwise; and (4) *visited doctor*, coded 1 if the responded reported having visited a physician in the last year and 0 otherwise. Table 1 presents the general summary statistics of our sample, grouped by *rural, semi-urban*, and *urban* locality.

### Statistical analyses

The regression analyses were performed using the R programming language for statistical computing, used two dependent variables, and the full set of covariates. The two models follow the specification outlined as follows:


(1) $$\begin{array}{l} \log{\left(medcost\right)}_{i}=\beta_{0}+\beta_{1}locality_{i}+\beta_{2}depression_{i}+\beta_{3}smoker_{i}\\ +X_{3}demographics_{i}+Y_{3}healthcare\_system_{i}+\in_{i} \end{array}$$

(2) $$\begin{array}{l} \log{\left(visitcost\right)}_{i}=\beta_{0}+\beta_{1}locality_{i}+\beta_{2}depression_{i}+\beta_{3}smoker_{i}+\\ X_{3}demographics_{i}+Y_{3}healthcare\_system_{i}+\in_{i} \end{array}$$

These models were then stratified by locality, where only the sample of each category (rural, urban, and semi-urban) was used. Table 2 shows the results of medication expenditures, while Table 3 shows the results of medical visits expenditures. Finally, a subsample (*n* = 349, 14.7%) of the population did not report any doctor visits in the previous year to the survey, warranting exploration in a different regression. For that reason, we added an additional regression that explored the relationships in the group without doctor visits, and *medication costs* as a variable of interest. For all these regressions, we did not include Social Security Institution in the healthcare variable, so that those respondents with any type of public or private insurance represent the baseline group and the coefficients for No Insurance and Seguro Popular represent the increased out-of-pocket expenditures of people who do not have insurance and fit into either of those categories. Regarding the interpretation of the results, the estimated coefficients were transformed into odds ratios, which represent the average effects at the mean of each predicted variable. For readability, 95% confidence intervals have been omitted, and for significance purposes, a *p*-value below 0.05 was used as the main threshold.

**Table 2:** Average effects of out-of-pocket medication expenses.

	**Medication expenses full**	**Medication expenses no visits**	**Medication expenses urban**	**Medication expenses semi-urban**	**Medication expenses rural**
**Demographics**					
Age	1.01	1.05	1.12	0.99	1.02^*^
Female	0.89	14.39^*^	0.55	1.21	1.11
Married	0.51^*^	0.36	0.74	0.55	0.55
Employed	0.62	1.58	2.08	0.84	0.67
Educational attainment (years)	1.12^*^	1.01	1.09^*^	1.08	1.25
**Locality**					
Semi-urban	0.57	0.92	-	-	-
Rural	1.05	0.25	-	-	-
**Health**					
Depressive symptoms	2.06^*^	1.37	2.15	2.73	1.81
Smoking	0.66	1.17	0.18^*^	1.65	0.43
Diabetes Medication	1.82	3.60	10.36	0.43	1.53
Insulin	1.29	0.31	5.28	1.47	0.62
**Health insurance** ^**++**^					
None	10.55^*^	16.87^*^	299.63^*^	36.4^*^	722.7^*^
Seguro Popular	3.97^*^	6.62^*^	1.96^*^	5.65^*^	20.35
**Use of services**					
physician visits	214.9^*^	-	-	-	-
N	2,368	349	1,480	577	311
Adjusted *R*^2^	0.168	0.06	0.065	0.02	0.038
F-statistic	32.08^*^	2.71^*^	10.44^*^	2.11^*^	2.10^*^

Significance level marker:

* *p* < 0.05.

^++^For health insurance, the reference category is respondents with employer-based insurance.

**Table 3:** Average effects of out-of-pocket medical visit expenses by locality.

	**Medical visit expenses**	**Medical visit expenses urban**	**Medical visit expenses semi-urban**	**Medical visit expenses rural**
**Demographics**				
Age	1.04^*^	1.02	1.05	1.13^*^
Female	1.51	1.24	2.85	1.22
Married	1.14	1.07	1.30	1.24
Employed	2.19^*^	1.29	5.27^*^	3.69
Educational attainment (years)	1.19^*^	1.14^*^	1.34^*^	1.23
**Locality**				
Semi-urban	1.42	-	-	-
Rural	1.69	-	-	-
**Health**				
Depressive symptoms	1.11	1.14	1.56	0.62
Smoking	0.67	0.54	1.03	0.70
Diabetes Medication	0.55	0.69	0.93	0.08
Insulin	0.57^*^	0.36^*^	0.97	1.66
**Health insurance** ^**++**^				
None	10,234^*^	7,521^*^	11,154^*^	26,020^*^
Seguro Popular	8.3^*^	5.25^*^	8.99^*^	21.62^*^
*N*	2,368	1,480	577	311
Adjusted R^2^	0.092	0.08	0.10	0.11
*F*-statistic	19.59^*^	12.75^*^	7.03^*^	4.44^*^

Significance level marker:

* *p* < 0.05.

^++^For Health Insurance, the reference category is respondents with employer-based insurance.

## Results

### Medication out-of-pocket expenditures

With regard to medication out-of-pocket expenditures as presented in Table 2, married respondents showed a statistically significant association, where being married was associated with a 49.1% average decrease in medication expenditures for the full sample. In the case of educational attainment, each additional year was associated with a 12% increase in medication expenses in the full sample and a 9% increase in medication expenses in the urban sample. The rest of the demographic variables (age, female, employed) did not have significant associations with medication expenses in the full sample, but female respondents did associate with a 1,339% average increase in medication expenses for the sample without medical visits, and age was significantly associated with a 2% increase in the rural sample.

With regard to locality, no significant associations were found for out-of-pocket medications expenses in any of the studied samples.

In terms of health variables, depressive symptoms were significantly associated with a 106% average increase in out-of-pocket medication expenditures in the full sample. With regard to smoking, taking diabetes oral medication, and insulin, only in the rural sample there was a statistically significant association, where smoking is associated with an 82% average decrease in out-of-pocket medication expenditures. With regard to health insurance, the group without health insurance was associated with a 955% increase in medication out-of-pocket expenditures compared with those in private or employment-based insurance, while those who participated in Seguro Popular were associated with a 297% average increase in medication expenses in the full sample. These increases hold their direction for the sample without medical visits, and the urban, semi-urban, and rural samples, except for Seguro Popular in the rural sample where no statistically significant association was found. Finally, visiting a physician was significantly associated with a dramatic increase in medication out-of-pocket expenditures in the full sample.

### Medical visits out-of-pocket expenditures

With regard to medical visit out-of-pocket expenditures as presented in Table 3, each additional year of age was significantly associated with a 4% increase in average medical visit expenditures in the full sample and 13% average increase in the rural sample. Being employed was significantly associated with a 119% increase in expenses for the full sample and 427% for the semi-urban sample. Every year of educational attainment was significantly associated with increases in medical visit expenditures. This increase was 19% for the full sample, 14% for the urban sample, and 34% for the semi-urban sample.

With regard to locality, we did not find any significant associations with changes in medical visit expenses. Insulin was significantly associated with lower medical visit expenditures in the full and urban samples, where it was associated with a 43% and 64% decrease, respectively.

For all the samples, not having insurance and participating in Seguro Popular were significantly associated with dramatic increases in medical visit expenditures.

## Discussion

This study offers significant insights into efforts to address Mexico's diabetes epidemic in that it explores the differences in out-of-pocket expenses in urban, semi-urban, and rural populations. Although Mexican legislation states that all Mexicans have a right to healthcare, the Mexican Health System is segmented and therefore access to, as well as the quality of care received, depends upon individual health insurance status and locality of residence, where in rural communities medical expenditures can take a significant portion of overall expenses (4). Recent news regarding the expansion of coverage for the uninsured, report insufficient resources for facilities, equipment physicians, ancillary health providers, drugs, and supplemental equipment for other adults. This means that in addition to a non-optimal system, incomplete coverage magnifies age–income-based disparities (34). Our findings may be partially explained by qualitative research in Mexico that attributes high expenditures to longer travel times to visit private providers because local public clinics are understaffed and do not provide efficient services (27, 35). This explanation, however, would only apply to medical visits and not medication out-of-pocket expenditures.

Regarding medications, high medication expenditures associated with depressive symptoms may be related to the medication needed to treat mental health ailments. Additionally, our findings highlight the difficulties observed in previous research with regard to accessing diabetes care medication in public clinics in rural communities in Mexico (26, 27).

Lower out-of-pocket expenditures for those affiliated with employment-based social security institutions may be as these insurance plans provide both medication and care for free at the point of services. In our sample, 52.7% of respondents in rural areas had access to Seguro Popular, compared with 38% of those in semi-urban communities and 15% in urban localities. Further, in rural locations where Seguro Popular has higher participation, medication out-of-pocket expenditures were much higher, possibly indicating difficulties for the program to provide its affiliates with all the medications they need. While time is needed to investigate the long-term effects of dissolving Seguro Popular and implementing INSABI, an evaluation by the National Council for Social Policy Evaluation (CONEVAL) notes that the number of Mexicans with healthcare insecurity, that is, those without access to health services, increased from 16.2 to 28.2 million from 2018 to 2020, while shortages of a wide variety of generic and specialized medicines have been documented nationwide (1). In both ways, these inequalities in out-of-pocket expenditures may increase and merit further investigation.

In addition, the results regarding demographic determinants of out-of-pocket expenditures deserve further study. It can be noted from Table 1 that being married, having lower educational attainment, and being female are characteristics more present in rural localities, which provides critical insights into health policy.

The dramatically higher association between being female and higher medication expenses in the no-visits group also deserves special attention. Gender differences in medication use have been found in many studies (36, 37). One of the main associated factors to this has been their higher use of health services compared with men, while in our study, women who did have medical visits had a much higher increased risk of medication expenditures. While these results merit future studies including other individual-level factors such as income, time with the chronic disease and taking medication, as well as detailed studies on medication use and polypharmacy, system-level factors that could be having an impact should also be included in future studies, for example, the direct link with the type of services the respondent has access to and the quality of services that could be causing individuals not approach services, but continue using/purchasing medicines previously prescribed, using pharmacies to obtain medical consultations and buying medicines which are not seen as part of the health system and therefore usually not reported as medical visits, among others. Finally, self-prescription, which has been reported as problematic in Mexico, could be also playing a role in such high expenditures and should be further investigated in this population group.

The female respondent result in the non-visit sample is an outlier in the analysis. The proportion of women and men in the no-visits group are similar (167 women and 182 men), and even if the distribution among other categories such as no insurance and participation in Seguro Popular is similar, this difference between expenditures cannot be accounted for. This drastic difference deserves further studies.

A limitation of the analysis is that any differences between medication and prescription drugs were not delineated in the reported data. As there is likely a price differential between over the counter and prescriptions as well as access, any differences between Seguro Popular provided medication and employer insurance should be a direction for future research.

Our data revealed that older adults with diabetes who are enrolled in Seguro Popular have higher out-of-pocket expenditures. Place-based disparities show that rural participants living with diabetes pay more than those in semi-urban and urban communities. Moreover, given the strong association between insulin and medical visit expenditures, we hypothesize that specialized care ends up translating to higher medical visit costs, especially for those in rural areas.

Previous studies find this income inequality in expenditures is associated with a lack of access to medication and services in the respondents' local clinics (27). Further attention is needed given the significant costs associated with access to specialty care for controlling diabetes. In future, the impact of these results on health, income, and family wellbeing should be investigated and integrated into regular evaluation of health and social policies and programs. Future studies should also examine gender disparities, due to women reporting lower levels of education, lower income, and a higher proportion of them lacked insurance. Specific policies seeking to address these gender disparities, like the ones implemented in areas such as educational attainment, employment, and gender-based violence, should be assessed.

In aging research, several studies have compared the limitations in access to care both in Mexico and using the US population as a comparison group (9, 31). Long-term care and cognitive impairment transitions have been studied, regarding expenses for hospitalization and nursing homes, with populations in the United States, where it has been found that higher out-of-pocket expenditures are associated with progressive declines in cognitive function and death. As a comparison, no acute long-term health cost burdens were found among older adults with normal cognition or for those who improved over the 8-year follow-up (38).

Similarly, a longitudinal study in Europe documented out-of-pocket expenses among older Israelis owing to lack of supplemental coverage. Costs of out-of-pocket spending are increasing among the most disadvantaged. Authors speculate that foreign born are more likely to pay out-of-pocket expenditures for healthcare. In Mexico, the equivalent to Part B Medicare coverage is extremely rare to cover 80% of out-of-pocket expenses as in the United States. In the Mexican context, INSABI pays for basic care, and little else. The access to private coverage for out-of-pocket expenditures is not universal, and this has significant policy implications (39).

A feedback loop in which higher rates of employment in semi-urban and urban areas compared with rural areas cause major differences in healthcare, in addition to lower cognitive functioning in rural areas which prevents employment, is suggested. An urban advantage associated with cognitive function has been documented using MHAS data (8). Overall, without insurance or adequate coverage, lower expenses may be associated with unmet need. As a result, individuals in rural communities are likely to have more difficulty in accessing adequate treatment for controlling their diabetes.

## Data availability statement

Publicly available datasets were analyzed in this study. This data can be founded at: http://www.mhasweb.org.

## Author contributions

AR contributed with the writing and draft of the paper, as well as the quantitative analysis, and the research design. CV, JA, and ML-O contributed with revisions of the draft of the paper, guiding the analysis, the research design, and the policy discussion. All authors contributed to the article and approved the submitted version.

## Funding

Support in funding for research provided by the University of Texas at Austin President's Award for Global Learning, 2020. Supplemental support was provided by NIH Grant #1R03AG063183-01.

## Conflict of interest

The authors declare that the research was conducted in the absence of any commercial or financial relationships that could be construed as a potential conflict of interest.

## Publisher's note

All claims expressed in this article are solely those of the authors and do not necessarily represent those of their affiliated organizations, or those of the publisher, the editors and the reviewers. Any product that may be evaluated in this article, or claim that may be made by its manufacturer, is not guaranteed or endorsed by the publisher.
